# Association of NPAR with 90-day mortality in predominantly acute-on-chronic liver failure

**DOI:** 10.3389/fmed.2026.1858263

**Published:** 2026-09-14

**Authors:** Jiahua Hu, Lu Li, Xiaoli Yang, Maohua Guo

**Affiliations:** Department of Infectious Diseases, The Third Affiliated Hospital of Chongqing Medical University, Chongqing, China

**Keywords:** 90-day mortality, liver failure, neutrophil percentage-to-albumin ratio (NPAR), prognosis, systemic inflammation

## Abstract

**Background:**

Acute-on-chronic liver failure (ACLF) carries high short-term mortality, and easily available prognostic biomarkers are urgently needed. The neutrophil percentage-to-albumin ratio (NPAR) integrates inflammatory and nutritional status, but its prognostic value in liver failure remains unclear. This study aimed to investigate the association between NPAR and 90-day mortality in patients with liver failure, hypothesizing that elevated NPAR would be associated with worse outcomes.

**Methods:**

This retrospective cohort study included 104 adult patients with liver failure (89.4% ACLF) admitted from January 2022 to December 2024. NPAR was calculated as neutrophil percentage divided by albumin concentration. The primary outcome was 90-day mortality. We used log-transformed NPAR in regression analyses to improve model stability. Multivariable logistic regression was performed with MELD score as the sole covariate in a parsimonious two-predictor model, with bootstrap validation (1,000 iterations). Formal tests for interaction were conducted to assess potential effect modification by sex, age (<60 vs. ≥60 years), and ascites status.

**Results:**

Of 104 patients, 29 (27.9%) died within 90 days. The non-survival group had significantly higher NPAR levels [median 2.51 (IQR: 2.17–2.82) vs. 1.88 (1.61–2.29), *P* < 0.001]. In univariate analysis, log-transformed NPAR was associated with mortality (OR: 2.67 per 1-unit increase in ln-NPAR, 95% CI: 1.04–6.86, *P* = 0.041). After adjustment for MELD score, the association was attenuated and no longer statistically significant (adjusted OR: 1.66, 95% CI: 0.40–6.82, *P* = 0.484). Bootstrap validation confirmed the instability of the adjusted estimate (median OR: 1.64, 95% CI: 0.45–9.73). Tests for interaction showed no significant effect modification by sex (*P*-interaction = 0.393), age (*P*-interaction = 0.419), or ascites status (*P*-interaction = 0.799).

**Conclusions:**

After adjustment for MELD score, no statistically significant independent association was demonstrated between ln-NPAR and 90-day mortality (adjusted OR: 1.66, 95% CI: 0.40–6.82). Whether NPAR provides incremental prognostic information beyond established severity scores remains to be determined in larger studies. These findings primarily apply to a predominantly ACLF population.

## 1 Introduction

Liver failure represents a severe clinical syndrome with high mortality rates, particularly within the critical first 90 days following diagnosis (1, 2). The complex interplay between liver dysfunction and systemic inflammation is increasingly recognized as a key factor in determining patient outcomes (3, 4). Identifying robust biomarkers that can reflect the inflammatory state and its association with mortality in liver failure is crucial for both clinical management and research purposes.

The neutrophil percentage-to-albumin ratio (NPAR), a readily available measure derived from routine blood tests, has been implicated in the pathogenesis of various inflammatory and neoplastic conditions (5, 6). It is hypothesized that NPAR may serve as a surrogate marker of systemic inflammation and immune dysregulation, potentially reflecting the severity of liver disease and its complications (7). Neutrophils play a pivotal role in the inflammatory response, and their increased percentage in the blood can indicate a heightened inflammatory state. This inflammatory response can exacerbate hepatic injury, promote multi-organ dysfunction, and contribute to hepatic encephalopathy. On the other hand, albumin is crucial for maintaining oncotic pressure, fluid balance, and transporting various substances. Hypoalbuminemia, often observed in liver failure, signifies poor nutritional reserves and impaired physiological defenses, which can compound the impact of systemic inflammation (8). Although previous studies have suggested a role for NPAR in the prognosis of liver cirrhosis, its relationship with 90-day mortality in patients with Acute-on-chronic liver failure (ACLF) and other forms of liver failure remains to be fully elucidated (9).

The hypothesis underlying this study is that NPAR, by integrating measures of inflammation and liver function, may provide a comprehensive index of the pathophysiological processes in liver failure. We propose that elevated NPAR levels could be indicative of a more severe inflammatory response and liver dysfunction, which could be associated with worse clinical outcomes. This study aims to investigate the association between NPAR and 90-day mortality in patients with liver failure, contributing to the understanding of the role of systemic inflammation in the prognosis of liver failure and offering a basis for future studies exploring the potential of NPAR as a prognostic indicator in this context.

## 2 Methods

### 2.1 Study design and population

This retrospective cohort study was conducted in the infectious diseases department from January 2022 to December 2024. Consecutive patients admitted to our department with a diagnosis of liver failure were considered for inclusion. All eligible consecutive patients during the study period (January 2022 to December 2024) were screened for inclusion. No formal sample-size calculation was performed *a priori*; the sample size was determined by the number of eligible patients available during the study period.

Inclusion criteria included: Adult patients aged 18 years or older; Hospitalized in the infectious diseases department for a minimum of 48 h to ensure adequate baseline assessment; Complete medical records available, including but not limited to complete blood count, liver function tests, renal function tests, and coagulation profiles.

Exclusion criteria included: Coagulation dysfunction due to reasons other than liver failure, such as disseminated intravascular coagulation (DIC); Pregnancy, to avoid the impact of pregnancy on liver function and coagulation; Missing key data (e.g., blood count data necessary for NPAR calculation), preventing complete analysis; Presence of other severe complications that could significantly affect prognosis, such as severe cardiovascular diseases or renal failure.

### 2.2 Definitions

The diagnosis and staging of liver failure were based on the guidelines from the Chinese Society of Infectious Diseases and the Chinese Society of Hepatology (10).

Liver failure is defined as the acute deterioration of liver function on the basis of chronic liver disease (with or without cirrhosis), accompanied by liver and/or extrahepatic organ failure (liver, kidney, brain, coagulation, respiratory, and circulatory systems) and a high short-term mortality rate, forming a complex clinical syndrome. The types of liver failure included: Acute liver failure (ALF), acute onset, no history of liver disease, within 4 weeks, characterized by hepatic encephalopathy of grade II or higher; Subacute liver failure (SALF), acute onset, no history of liver disease, within 4–24 weeks, with clinical manifestations of liver failure; Acute-on-chronic (subacute) liver failure (ACLF), on the basis of chronic liver disease, various factors lead to acute liver function deterioration within a short period, characterized by liver and/or extrahepatic organ failure and a high short-term mortality rate; Chronic liver failure (CLF), on the basis of cirrhosis, slowly progressive liver function impairment leading to recurrent ascites and/or hepatic encephalopathy as the main manifestations of chronic liver function decompensation (10).

The staging of liver failure (early, middle, late) was defined according to the 2024 Guidelines for the Diagnosis and Treatment of Liver Failure by the Chinese Society of Infectious Diseases and the Chinese Society of Hepatology (10):

Early stage: Severe jaundice [total bilirubin (TB) ≥ 10× upper limit of normal or rapid daily increase ≥17.1 μmol/L] with no obvious coagulation disorder [prothrombin activity (PTA) >40% or international normalized ratio (INR) <1.5], and no hepatic encephalopathy.

Middle stage: Progression from early stage with worsening coagulation disorder (PTA: 30%–40% or INR: 1.5–1.9) and/or grade II hepatic encephalopathy, without obvious ascites or infection.

Late stage: Refractory complications including severe coagulation disorder (PTA < 30% or INR ≥ 2.0), grade II or higher hepatic encephalopathy, and/or multiple organ failure (hepatic, renal, circulatory, respiratory).

### 2.3 Data collection

Demographic data, clinical characteristics, and laboratory parameters were collected from patients' medical records. In cases where patients experienced significant fluctuations in their laboratory parameters within the first 24 h of hospitalization, we recorded the worst values during this period. These included age, gender, white blood cell (WBC) count, neutrophil count, lymphocyte count, platelet count (PLT), red blood cell count (RBC), hemoglobin (Hb), international normalized ratio (INR), fibrinogen (Fib), alanine aminotransferase (ALT), aspartate aminotransferase (AST), alkaline phosphatase (ALP), gamma-glutamyl transferase (GGT), albumin (ALB), globulin (GLB), total bilirubin (TB), direct bilirubin (DB), serum creatinine, and NPAR. NPAR was calculated for each patient using the formula: NPAR = Neutrophil percentage/ALB.

MELD score was calculated using the standard formula: MELD = 3.78 × ln[serum bilirubin (mg/dL)] + 11.2 × ln[INR] + 9.57 × ln[serum creatinine (mg/dL)] + 6.43. For patients with creatinine <1.0 mg/dL, a value of 1.0 was used. For missing laboratory values, we used the worst value within the first 24 h of hospitalization. Patients with missing data necessary for MELD calculation were excluded. For NPAR calculation, patients with missing neutrophil percentage or albumin were excluded (*n* = *X*). For other variables, missing data were handled by complete-case analysis.

### 2.4 Statistical analysis

Statistical analyses were performed using R version 4.2.3. Continuous variables are presented as median (interquartile range, IQR) and compared using the Mann–Whitney *U* test. Categorical variables are presented as frequencies (percentages) and compared using the chi-squared or Fisher's exact test.

Given the right-skewed distribution of NPAR, we used natural log-transformation (ln-NPAR) for regression analyses to reduce the influence of extreme values and improve model convergence. Univariate logistic regression was performed to assess the association between ln-NPAR and 90-day mortality. For multivariable analysis, we first applied LASSO (Least Absolute Shrinkage and Selection Operator) regression with 5-fold cross-validation for variable selection. To avoid overfitting, we restricted the final multivariable model to no more than 2–3 predictors, following the rule of at least 10 outcome events per predictor variable (EPV ≥ 10). Given that MELD score is the established standard for liver disease severity prognostication, we included it as a mandatory covariate in the primary multivariable model. Bootstrap resampling with 1,000 iterations was performed to assess the stability of odds ratio estimates. Bias-corrected accelerated (BCa) confidence intervals were calculated. We assessed potential effect modification by sex, age (<60 vs. ≥60 years), and ascites status using formal tests for interaction. Stratified odds ratios were not reported due to limited sample size and small numbers of outcome events in several subgroups.

A two-sided *P*-value <0.05 was considered statistically significant.

## 3 Results

### 3.1 Clinical characteristics

A total of 127 patients with liver failure were initially identified from electronic medical records during the study period (January 2022 to December 2024). Of these, 23 patients were excluded based on predefined criteria, resulting in a final analytic cohort of 104 patients with 75 patients in the survival group and 29 in the non-survival group (Figure 1). Among the 23 excluded patients, none were excluded solely due to death within 48 h of admission. The exclusions were based on the predefined criteria. The stage of liver failure was a strong factor, with advanced stages being more prevalent in non-survivors (*P* < 0.001). The presence of ascites (*P* < 0.001) and liver cirrhosis (*P* < 0.001) were also significantly increased in non-survival group. There was no difference in gender distribution between the two groups (*P* = 0.380).

**Figure 1:**
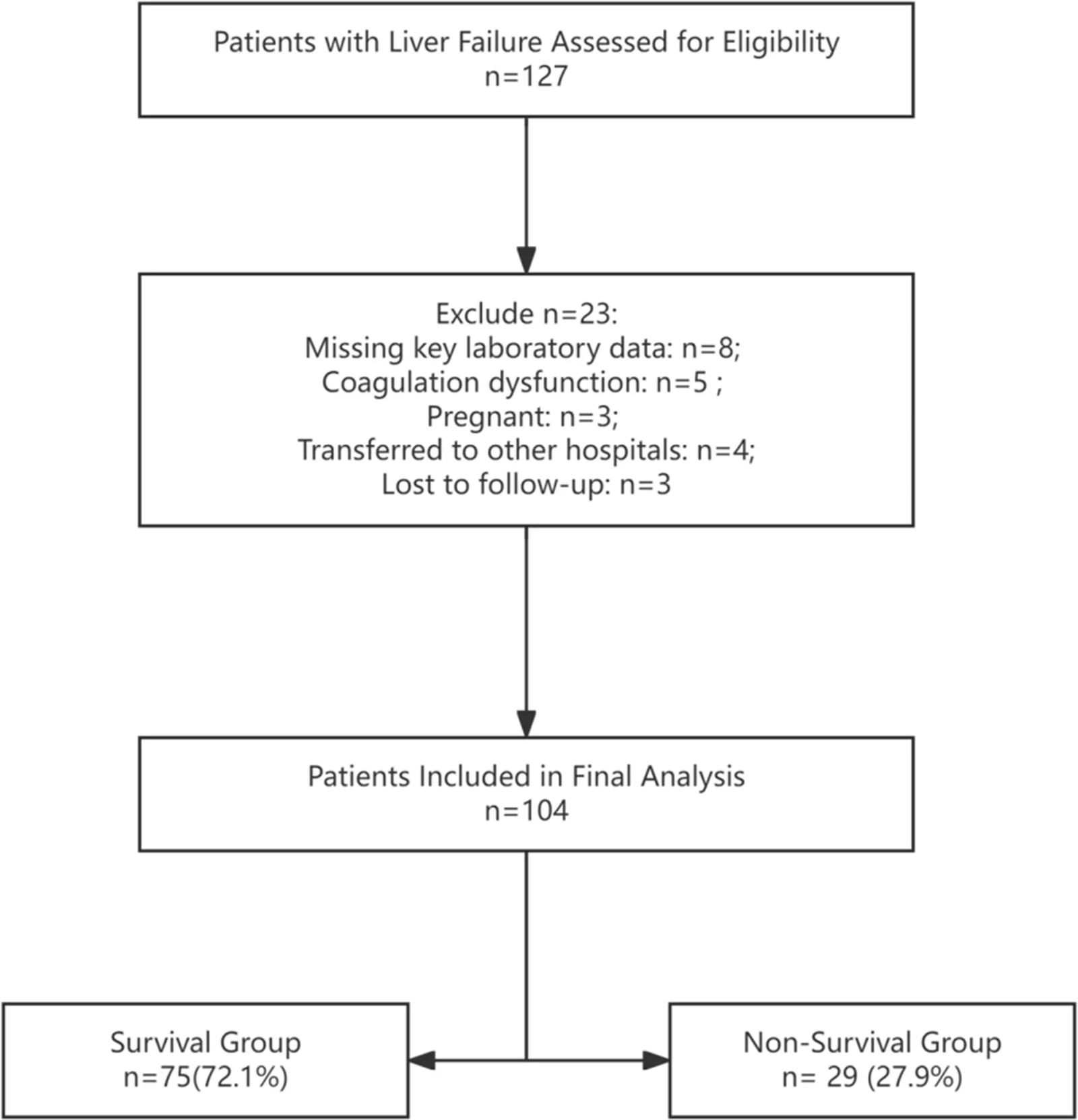
Patient selection flow diagram.

Laboratory findings revealed that the non-survival group had significantly lower Lym count, PLT, RBC, Hb, Fib, ALT, AST, GGT, and ALB. Additionally, higher Neu count, INR, TB, DB and NPAR were observed in the non-survival group (Table 1).

**Table 1:** Baseline characteristics and comparisons between survival and non-survival groups.

Variables	Total (*n* = 104)	Survival group (*n* = 75)	Non-survival group (*n* = 29)	Statistic	*P*
Age, M (Q_1_, Q_3_)	46.00 (35.75, 56.00)	44.00 (33.50, 54.50)	54.00 (42.00, 58.00)	*Z* = −2.36	0.018
Stages, *n* (%)				*χ*^2^ = 63.28	<0.001
Early stage	69 (66.35)	66 (88.00)	3 (10.34)		
Middle stage	22 (21.15)	9 (12.00)	13 (44.83)		
Late stage	13 (12.50)	0 (0.00)	13 (44.83)		
Gender, *n* (%)				*χ*^2^ = 0.77	0.380
Male	80 (76.92)	56 (74.67)	24 (82.76)		
Female	24 (23.08)	19 (25.33)	5 (17.24)		
Ascites, *n* (%)				*χ*^2^ = 27.31	<0.001
No	57 (54.81)	53 (70.67)	4 (13.79)		
Yes	47 (45.19)	22 (29.33)	25 (86.21)		
Liver cirrhosis, *n* (%)				*χ*^2^ = 44.98	<0.001
No	67 (64.42)	63 (84.00)	4 (13.79)		
Yes	37 (35.58)	12 (16.00)	25 (86.21)		
Types of liver failure, *n* (%)				—	0.020
ACLF	93 (89.42)	65 (86.67)	28 (96.55)		
ALF	1 (0.96)	0 (0.00)	1 (3.45)		
SALF	10 (9.62)	10 (13.33)	0 (0.00)		
WBC, M (Q_1_, Q_3_)	5.84 (4.68, 7.28)	5.77 (4.71, 7.22)	6.53 (4.60, 7.31)	*Z* = −0.43	0.664
Neu count, M (Q_1_, Q_3_)	3.80 (3.27, 5.34)	3.59 (3.08, 5.03)	4.48 (3.62, 5.66)	*Z* = −1.99	0.046
Lym count, M (Q_1_, Q_3_)	1.11 (0.67, 1.49)	1.23 (0.80, 1.53)	0.70 (0.56, 1.35)	*Z* = −2.40	0.016
PLT, M (Q_1_, Q_3_)	119.50 (66.75, 172.25)	140.00 (87.50, 194.00)	61.00 (48.00, 74.00)	*Z* = −4.95	<0.001
RBC, M (Q_1_, Q_3_)	4.33 (3.67, 4.71)	4.44 (4.11, 4.78)	3.67 (2.77, 4.12)	*Z* = −2.95	0.003
Hb, M (Q_1_, Q_3_)	134.00 (115.75, 150.25)	141.00 (125.00, 152.00)	122.00 (99.00, 134.00)	*Z* = −3.38	<0.001
INR, M (Q_1_, Q_3_)	1.73 (1.44, 2.44)	1.57 (1.37, 1.90)	3.19 (2.23, 3.90)	*Z* = −6.01	<0.001
Fib, M (Q_1_, Q_3_)	1.58 (1.21, 2.11)	1.72 (1.42, 2.20)	1.19 (0.90, 1.74)	*Z* = −3.82	<0.001
ALT, M (Q_1_, Q_3_)	703.00 (137.75, 1,730.25)	949.00 (303.50, 1,794.00)	155.00 (44.00, 1,070.00)	*Z* = −2.76	0.006
AST, M (Q_1_, Q_3_)	540.50 (140.25, 987.00)	556.00 (228.50, 1,088.00)	141.00 (95.00, 765.00)	*Z* = −2.42	0.015
ALP, M (Q_1_, Q_3_)	147.00 (113.50, 198.75)	148.00 (121.00, 202.50)	131.00 (98.00, 180.00)	*Z* = −1.38	0.167
GGT, M (Q_1_, Q_3_)	121.00 (71.50, 232.75)	126.00 (89.50, 251.00)	90.00 (41.00, 146.00)	*Z* = −2.97	0.003
ALB, M (Q_1_, Q_3_)	34.40 (29.45, 38.65)	35.90 (31.70, 40.65)	30.20 (27.30, 34.50)	*Z* = −4.05	<0.001
GLB, M (Q_1_, Q_3_)	31.70 (27.07, 34.85)	31.50 (27.00, 33.35)	34.10 (28.70, 39.65)	*Z* = −1.56	0.118
TB, M (Q_1_, Q_3_)	234.45 (134.60, 338.10)	177.00 (126.05, 270.55)	354.80 (280.80, 439.50)	*Z* = −4.85	<0.001
DB, M (Q_1_, Q_3_)	148.05 (93.90, 227.40)	138.50 (78.90, 188.75)	216.90 (165.10, 281.20)	*Z* = −3.49	<0.001
Serum creatinine, M (Q_1_, Q_3_)	69.00 (59.00, 93.75)	72.00 (60.00, 96.00)	67.00 (58.00, 82.00)	*Z* = −1.63	0.103
NPAR, M (Q_1_, Q_3_)	2.05 (1.74, 2.48)	1.88 (1.61, 2.29)	2.51 (2.17, 2.82)	*Z* = −4.76	<0.001
MELD score, M (Q_1_, Q_3_)	22.4 (19.2, 27.1)	20.0 (18.2, 23.5)	31.5 (28.9, 33.1)	*Z* = −6.85	<0.001

*Z*, Mann–Whitney test; *χ*^2^, Chi-square test; —, Fisher exact; M, Median; Q_1_, 1st Quartile; Q_3_, 3st Quartile.

PLT, Platelet Count (10^9^/μL); RBC, Red Blood Cell Count (10^12^/L); Hb, Hemoglobin (g/L); INR, International Normalized Ratio; Fib, Fibrinogen (g/L); ALT, Alanine Aminotransferase (U/L); AST, Aspartate Aminotransferase (U/L); ALP, Alkaline Phosphatase (U/L); GGT, Gamma-Glutamyl Transferase (U/L); ALB, Albumin (g/L); GLB, Globulin (g/L); TB, Total Bilirubin (μmol/L); DB, Direct Bilirubin (μmol/L); Serum Creatinine (μmol/L); NPAR, Neutrophil percentage/Albumin ratio.

### 3.2 Association between NPAR and 90-day mortality in predominantly ACLF patients

In univariate logistic regression, log-transformed NPAR was significantly associated with 90-day mortality (OR: 2.67 per 1-unit increase, 95% CI: 1.04–6.86, *P* = 0.041) (Table 2). Given the limited number of outcome events (*n* = 29), we restricted the multivariable model to two predictors to maintain adequate events per variable (EPV = 14.5). We selected MELD score as the covariate because it is the established standard for prognostication in liver disease. The multivariable model therefore included log-transformed NPAR and MELD score only.

**Table 2:** Univariate and multivariate logistic analysis for 90-day mortality.

Variables	Univariate analysis			Multivariable analysis*		
	*β*	OR (95% CI)	*P*	*β*	OR (95% CI)	*P*
ln-NPAR (per 1-unit increase)	0.98	2.67 (1.04–6.86)	0.041	0.51	1.66 (0.40–6.82)	0.484
MELD score (per 1-point increase)	0.48	1.62 (1.35–1.94)	<0.001	0.47	1.60 (1.34–1.93)	<0.001

* Multivariable model included ln-NPAR and MELD score only, restricted to two predictors to maintain adequate events per variable (EPV = 14.5). ln-NPAR was calculated as the natural logarithm of NPAR (neutrophil percentage/albumin in g/dL). In regression analyses, the OR for ln-NPAR represents the change in odds of 90-day mortality associated with a 1-unit increase in ln-NPAR (approximately a 2.72-fold increase in raw NPAR). Bootstrap validation with 1,000 iterations confirmed the instability of the adjusted ln-NPAR estimate (median OR: 1.64, 95% CI: 0.45–9.73).

In the multivariable model adjusting for MELD score, the association between log-transformed NPAR and mortality was attenuated and no longer statistically significant (adjusted OR: 1.66, 95% CI: 0.40–6.82, *P* = 0.484). MELD score remained strongly associated with mortality (adjusted OR: 1.60, 95% CI: 1.34–1.93, *P* < 0.001) (Table 2). Bootstrap validation with 1,000 resamples confirmed the instability of the adjusted NPAR estimate (median OR: 1.64, bootstrap 95% CI: 0.45–9.73), with successful convergence in all iterations.

### 3.3 Tests for interaction by sex, age, and ascites status

Formal tests for interaction were conducted to assess potential effect modification by sex, age (<60 vs. ≥60 years), and ascites status. No significant effect modification was observed for sex (*P*-interaction = 0.393), age (*P*-interaction = 0.419), or ascites (*P*-interaction = 0.799). Stratified odds ratios were not reported due to limited sample size and small numbers of outcome events in several subgroups. These findings do not support differential prognostic utility of NPAR across these subgroups (Table 3).

**Table 3:** Tests for interaction between NPAR and 90-day mortality in subgroups of liver failure patients.

Subgroup	Category	Events/total	Interaction *P*-value
Sex	Male	24/80	0.393
	Female	5/24	
Age, years	<60	19/75	0.419
	≥60	10/29	
Ascites	No	4/57	0.799
	Yes	25/47	

Stratified odds ratios are not reported for subgroups with fewer than 5 outcome events due to insufficient statistical power. Formal tests for interaction showed no significant effect modification by any subgroup.

## 3 Discussion

This retrospective study of 104 patients with liver failure (89.4% ACLF) found that NPAR was significantly higher in non-survivors and was associated with 90-day mortality in univariate analysis. However, after adjustment for MELD score—a well-validated prognostic index in liver disease—the association was attenuated and no longer statistically significant. These findings suggest that the prognostic information captured by NPAR may overlap substantially with that contained in established severity scores such as MELD, which was originally developed for cirrhosis patients undergoing TIPS (11) and has recognized limitations in ACLF assessment (12).

Log-transformed NPAR (ln-NPAR) was significantly associated with 90-day mortality in univariate analysis. However, after adjustment for MELD score, the association for ln-NPAR was attenuated and no longer statistically significant, suggesting that the prognostic information captured by NPAR overlaps substantially with disease severity as measured by established scoring systems. The higher NPAR values in the non-survival group may reflect a more pronounced inflammatory response, which could contribute to the worsening of liver function and the development of complications that lead to mortality (13). Our findings are in line with previous studies that have identified NPAR as a prognostic factor in various conditions, including Cirrhosis and fatty liver (8). A recent report also indicates that NPAR is independently associated with a higher risk of death in patients with advanced chronic liver disease (14). However, our finding that NPAR lost significance after MELD adjustment differs from these reports. Du et al. (9) found that NPAR predicted mortality independently of Child-Pugh and MELD scores in patients with liver cirrhosis. Their study differed from ours in several important respects: they included compensated and decompensated cirrhosis rather than acute liver failure; their outcome was long-term mortality rather than 90-day mortality; and their sample size was substantially larger, providing greater statistical power to detect modest independent effects. In contrast, our study used log-transformed NPAR in a parsimonious two-predictor model with MELD as the sole covariate, reflecting our limited sample size and event count (*n* = 104, 29 deaths). These differences in population, outcome period, and statistical precision limit direct comparability. Within our cohort, NPAR's lack of independent prognostic value likely reflects its pathophysiological overlap with MELD: albumin and neutrophil percentage capture synthetic function and inflammation that are also partially represented in MELD components. In our predominantly ACLF population, short-term mortality is driven by acute organ dysfunction, leaving limited incremental information for NPAR beyond MELD. This contrasts with cirrhosis, where persistent inflammation over longer periods may allow NPAR to capture prognostic information not fully reflected in a single severity score. These considerations apply specifically to our predominantly ACLF cohort and should not be generalized to other liver failure subtypes (ALF, SALF, CLF) without further validation in more diverse cohorts.

The elevated NPAR levels could be indicative of a systemic inflammatory response syndrome, which is known to be associated with poor outcomes in critical illness (15). In the context of liver failure, this inflammatory response may be driven by the release of pro-inflammatory cytokines from the damaged liver, leading to a shift in the balance between neutrophils and lymphocytes (16). In addition to NPAR, other inflammatory markers, including the neutrophil-to-lymphocyte ratio and red blood cell distribution width, have been independently associated with 90-day mortality in patients with ACLF (17). Importantly, because NPAR is proposed as an inflammation-based biomarker, the absence of detailed infection and inflammatory data represents a critical limitation. Our retrospective dataset did not include systematic documentation of infection status at admission (e.g., confirmed sepsis, spontaneous bacterial peritonitis, pneumonia), dynamic inflammatory biomarkers (e.g., C-reactive protein, procalcitonin), or major treatment variables (e.g., antibiotic regimens, corticosteroid use). In liver failure, infection is a well-established trigger of systemic inflammation and a major determinant of neutrophil response and short-term mortality (18). Similarly, pro-inflammatory cytokine release from the damaged liver drives neutrophilia and correlates with prognosis (16). Consequently, residual confounding from these unmeasured variables cannot be excluded, and the observed univariate association between NPAR and mortality may partly reflect underlying infection severity and treatment intensity rather than an independent biological effect of NPAR itself. Future studies should prospectively collect comprehensive infection, inflammation, and treatment data to disentangle these relationships.

The observed associations between elevated NPAR and mortality may be underpinned by the following mechanisms. Elevated neutrophil percentages are indicative of an enhanced inflammatory response, which exacerbates hepatic injury, promotes multi-organ dysfunction, and contributes to hepatic encephalopathy (18). Albumin serves crucial anti-inflammatory, antioxidant, and transport functions. Low albumin levels signify poor nutritional reserves and impaired physiological defenses, compounding the impact of systemic inflammation (19). The imbalance of NPAR reflects a state of immune dysfunction, where neutrophil overactivation and reduced albumin levels impair host defense mechanisms, increasing susceptibility to complications (20).

Subgroup analyses showed no significant effect modification by sex, age, or ascites status (all *P*-interaction > 0.05). Due to limited sample size and small numbers of outcome events in several subgroups, stratified odds ratios were not reported. These findings do not support differential prognostic utility of NPAR across these subgroups.

Our retrospective analysis indicates that ln-NPAR is associated with 90-day mortality in univariate analysis, but no statistically significant independent association was demonstrated after adjustment for MELD score. These findings suggest that NPAR's prognostic value in this cohort may overlap with disease severity as measured by established scoring systems, but the wide confidence interval (0.40–6.82) indicates considerable imprecision, and a true independent effect cannot be excluded.

Several limitations should be considered. First, the retrospective single-center design may introduce selection and measurement bias. Second, the small sample size (*n* = 104, 29 deaths) and the resulting imprecision of the adjusted estimate (95% CI: 0.40–6.82) limit our ability to exclude a modest independent effect of NPAR. Third, the predominance of ACLF cases (93 of 104 patients) restricts generalizability to other liver failure subtypes. Fourth, residual confounding from unmeasured or incompletely captured variables—including infection, sepsis, spontaneous bacterial peritonitis, other inflammatory markers, and treatment variables, cannot be excluded. Fifth, we were unable to formally evaluate the incremental predictive value of NPAR beyond MELD using metrics such as ROC/AUC comparison, net reclassification improvement (NRI), or integrated discrimination improvement (IDI) because the small sample size and limited event count render such analyses statistically unreliable and prone to overfitting; we explicitly acknowledge this as a major methodological limitation. Sixth, we acknowledge the risk of confirmation bias: the significant univariate association and apparent subgroup differences, though not statistically significant after adjustment or interaction testing, may have been emphasized in our interpretation. Finally, the lack of external validation and the absence of a formal sample-size calculation limit the robustness and generalizability of our findings. We are planning a prospective multicenter study to address these limitations. Future studies should employ prospective multicenter recruitment, prespecified sample-size planning, and standardized baseline measurement. Comprehensive collection of infection and treatment variables, including sepsis, spontaneous bacterial peritonitis, and antibiotic use, would address residual confounding. External validation in independent cohorts and formal comparison of discrimination, calibration, and clinical utility against established prognostic scores are essential before NPAR can be considered for clinical application.

We are planning a prospective multicenter study to address these limitations. Despite these limitations, our findings are hypothesis-generating and warrant further investigation. Future studies should employ prospective multicenter recruitment, prespecified sample-size planning, and standardized baseline measurement. Comprehensive collection of infection and treatment variables—including sepsis, spontaneous bacterial peritonitis, and antibiotic use—would address residual confounding. External validation in independent cohorts and formal comparison of discrimination, calibration, and clinical utility against established prognostic scores are essential before NPAR can be considered for clinical application.

## 4 Conclusion

In this retrospective cohort of 104 patients with liver failure (89.4% ACLF), log-transformed NPAR (ln-NPAR) was associated with 90-day mortality in univariate analysis, but no statistically significant independent association was demonstrated after adjustment for MELD score (adjusted OR: 1.66, 95% CI: 0.40–6.82). The wide confidence interval indicates substantial imprecision, and whether NPAR provides incremental prognostic information beyond established severity scores remains undetermined. These findings primarily apply to a predominantly ACLF population and should not be generalized to other liver failure subtypes without further validation. These hypothesis-generating findings require confirmation in larger, prospective, multicenter studies with comprehensive collection of infection and treatment variables.

## Data Availability

The original contributions presented in the study are included in the article/Supplementary Material, further inquiries can be directed to the corresponding author.
